# Toxicity Analysis of Some Frequently Used Food Processing Chemicals Using *Allium cepa* Biomonitoring System

**DOI:** 10.3390/biology12050637

**Published:** 2023-04-22

**Authors:** Md. Shimul Bhuia, Md. Sajjad Hossain Siam, Md. Riat Ahamed, Uttam Kumar Roy, Md. Imran Hossain, Md. Rokonuzzman, Tawhida Islam, Rezoan Sharafat, Mehedi Hasan Bappi, Md. Nayem Mia, Md. Emamuzzaman, Ray Silva de Almeida, Henrique Douglas Melo Coutinho, António Raposo, Hmidan A. Alturki, Muhammad Torequl Islam

**Affiliations:** 1 Department of Pharmacy, Bangabandhu Sheikh Mujibur Rahman Science and Technology University, Gopalganj 8100, Bangladesh; 2 Department of Biological Chemistry, Regional University of Cariri—URCA, Crato 63105-000, CE, Brazil; 3 CBIOS (Research Center for Biosciences and Health Technologies), Universidade Lusófona de Humanidades e Tecnologias, Campo Grande 376, 1749-024 Lisboa, Portugal; 4 General Directorate for Funds & Grants, King Abdulaziz City for Science & Technology, Riyadh 11442, Saudi Arabia

**Keywords:** toxic effect, formalin, saccharin, urea, eukaryotic system

## Abstract

**Simple Summary:**

This study showed that the chemical agents (FML, SCN, and urea) used in food processing produced mild concentrations and exposure time-dependent toxicity in *A. cepa*. The chemical agents exhibited time-dependent protective effects up to 72 h inspection of 24 h, and depletion of %RG was observed at 72 h inspection of 48 h. Our study suggests that adequate precautions and minimization of doses are needed in the case of industrial and local usage of these chemicals, as the chemicals produced concentration-dependent toxicity in this eukaryotic test system.

**Abstract:**

Frequent use of various food processing chemical agents sometimes causes damage to our bodies by inducing cytotoxicity, genotoxicity, and mutagenesis. In Bangladesh, among various chemical agents, formalin, saccharin, and urea are vastly used for processing foodstuffs by industry and local people. This study is focused to assess the toxic effects of formalin, saccharin, and urea on the popularly used eukaryotic test model, *Allium cepa* L. The assay was carried out by exposing different concentrations of test samples to *A. cepa* at 24, 48, and 72 h, where distilled water and CuSO_4_·5H_2_O (0.6 µg/mL) were utilized as the vehicle and positive control, respectively. The root length of the onions was measured in mm, and the results propose that all the chemical agents demonstrated toxicity in onions in a concentration- and exposure-time-dependent manner. The highest root length was examined at the lower concentrations, and with the increase in the concentration of the test sample and exposure time, the RG (root growth) was inhibited due to the deposition of chemicals and hampering of cell division in the root meristematic region of *A. cepa*. All the chemical agents also revealed a concentration- and time-dependent adaptive effect up to 72 h inspection of 24 h and a depletion of % root growth at 72 h inspection of 48 h. Our study suggests that sufficient precautions should be confirmed during its industrial and traditional usage as a toxicological response to the chemical agents observed in the *A. cepa* assay.

## 1. Introduction

Toxicity analysis is crucial for the potential risk assessment of a wide variety of substances, including drugs and chemicals. The preliminary toxicity investigation of various biological systems demonstrates the inquiring compound’s species-, organ-, and dose-dependent toxic effects. The toxicities of a compound can be accomplished by performing different procedures, such as (a) investigating the fortuitous disclosures to a compound, (b) in vitro analysis utilizing cells or cell lines, and (c) in vivo studies using laboratory animals [1]. The elemental purpose of toxicological investigation in the drug discovery and development process is to appraise the safety of a drug candidate [1] and identify the probable adverse effects that occur by the candidate, for example, genotoxicity, immunotoxicity, carcinogenicity, etc., which are particularly evident after chronic exposure to that chemical [2].

The *Allium (Allium cepa)* test is a well-organized experimental method for chemical compound screening and in situ monitoring for the cytogenotoxicity of varieties of substances, including crude extracts, isolated natural compounds, laboratory derivatives, environmental toxicants, etc. The test procedure has been vastly utilized to investigate DNA damages, such as chromosome aberrations and disturbances in the mitotic cycle in the root meristems of *A. cepa* caused by toxic agents [3,4]. The test system is described as a cost-effective procedure and easy to handle, as well as advantageous over other short-term test methods that prerequisite the previous preparation of experimented samples [5].

The human body cannot do without food. Various chemical agents are frequently used in food processing or by local people to store or cook foods worldwide; for example, certain preservatives and food additives (e.g., aspartame, carrageenan, sodium benzoate, vitamin A, tartrazine, and potassium benzoate). Large doses or chronic use of these chemical substances are evidently linked to many diseases and disorders, such as obesity, asthma, cancer, and neurological and cardiac diseases and disorders [6]. At present, food security is one of the major concerns in Bangladesh (a developing South Asian country) [7]. Many illiterate and inexperienced people who process foods for small- and large-scale supplies usually use numerous chemicals during food processing and preservation. Among these chemicals, formalin (FML) is widely used to preserve different foodstuffs, such as vegetables, fruits, and fish, which keeps the bodies of these foods seemingly fresh and solid by impersonating internal festering, and they are openly sold in local and supermarkets [8]. Studies have shown that direct consumption of FML is the most threatening to the human body as it may cause cancer, especially liver and lung cancer and cirrhosis [9]. It is also observed that the ingestion of a minute amount of FML may be responsible for corrosive damage to the GI mucosa, with nausea, vomiting, bleeding, perforation, and pain, as well as harmful systemic effects, including metabolic acidosis, coma, CNS depression, renal failure, and respiratory failure [10]. Even individuals who inject or spray FML run the risk of developing health issues such as blindness or asthma as a result of doing so repeatedly over a prolonged period of time [11]. According to the US Environmental Protection Agency (EPA), the maximum daily dose reference (RfD) for formaldehyde is 0.2 mg/kg of body weight per day. However, the intake of FML in food processing is prohibited in Bangladesh to avoid the hazardous effects of this chemical [12].

Nowadays, artificial sweeteners are used as a very popular food additive and as a sugar substitute in food processing. Among them, saccharin (SCN) is a high-intense sweetener that is widely utilized in the food, confectionery, beverage, and pharmaceutical industries [13]. It is presently granted as safe for intake by the FDA and may be utilized in beverages, drinks, fruit juice drinks, and bases or mixes, in accordance with guidance as a sugar substitute for cooking or table use, and in commercially prepared foods at a dose of 15 mg/kg/day (the FDA). Besides its beneficial utilization, it has exhibited significant toxicity in the bodies of experimental animals, as well as humans [14]. A recent investigation demonstrated that long-time utilization of SCN enhances glucose intolerance by affecting gut microorganisms, resulting in prediabetic consequences, such as insulin resistance, heart disease, stroke, obesity, and mortality risk [15,16]. In large quantities, SCN added to foods or consumption, especially above 1 gm for a long period of time, is liable to induce disturbances of digestion [17] and promote liver inflammation [14].

On the other hand, urea is a vast source of nitrogen in agriculture and is used as a fertilizer and an animal feed additive [18]. In the human body, urea is produced by the protein metabolism in the liver and kidneys through the ornithine cycle by combining two ammonia molecules (NH_3_) with a carbon dioxide (CO_2_) molecule and is later excreted as a constituent of urine [19]. Though it is utilized as fertilizer, in Bangladesh, it is also used as a food processing agent in rice factories to increase the elegance of rice by polishing and in the production of parched rice (locally known as Muri). According to the European Chemical Agency (ECHA), urea is a non-toxic (LD_50_ is 15 g/kg for rats) chemical within an optimum dose, and the approved dose for urea intake is 250 mg/kg/day. An excessive amount of urea intake may cause tissue damage, especially in the liver and kidneys, as well as dermatitis [20].

Knowing the above-mentioned facts, we aimed to investigate the toxic effects of FML, SCN, and urea using the eukaryotic test model, *Allium cepa*.

## 2. Materials and Methods

### 2.1. Collection of Allium cepa, Test Samples, or Standard

Medium-sized fresh onions (*A. cepa*) were collected from a local vegetable store in Pabna, Bangladesh, in 2022. Formalin (FML), saccharin (SCN), and copper sulfate were purchased from Merck (India), and urea was purchased from Chittagong Urea Fertilizer Ltd. Distilled Water (Vehicle), and other adjuvant instruments required for this investigation were purchased from the local market of Pabna, Bangladesh.

### 2.2. Selection and Preparation of Test Concentrations

The concentrations of the test samples were chosen on the basis of a literature review. We took three concentrations of each test sample (one lower and one higher than the approved dose). All the test samples and standard (copper sulfate) were vigorously soluble in water (DW). The solid crystals of copper sulfate, SCN, urea, and liquid FML were dissolved in DW with manual intermittent shaking for about 30 min to prepare the highest concentration, which was referred to as the mother solution (MS). The MS was then diluted by DW to procure the required concentrations of each test sample.

### 2.3. Study Protocol

The outer layers and budding parenchyma of clean and healthy onion bulbs were cautiously dispelled by forming a tiny circular incision to assist root growth (RG). The bulbs were then washed with distilled water for 10 min, and the root portion was placed in cleaned glass containers containing DW (capacity: 8–10 mL) for the first 24 h at room temperature in a shadowy place to generate roots. The bulbs with sufficient good RG were moved to the container (four for each concentration of test samples and five for control groups), remaining the sample/control for the exposure of 24, 48, and 72 h. After the exposure period, the length of the roots (15 roots, which are the longest) was measured in mm and noted every 24 h up to 72 h. The volume of the samples/control was adjusted every 24 h with the help of a dropper. %IRG and IC_50_ were also calculated for the test samples. Copper sulfate (0.6 µg/mL) and DW were utilized as positive and negative controls, respectively.

### 2.4. Statistical Analysis

GraphPad Prism (version 6.0) was used to analyze the data, which included an analysis of variance (one-way ANOVA) followed by a Tukey post hoc test, with *p* < 0.05 and a confidence level of 95%, and the values are presented as the mean ± standard error of the mean (SEM) or percentage.

## 3. Results

In the *A. cepa* assay, a concentration- and exposure-time-dependent toxic effect was demonstrated. *A. cepa* treated with different concentrations of a food processing chemical named formalin (a 40% solution of formaldehyde in water) at various exposure times (24, 48, and 72 h) revealed a variation in the mean of the root length due to toxicity. The mean of the root length of the onions treated with FML and exposed at 24, 48, and 72 h is exhibited in Table 1. The highest RG was found in the test group (con. 0.10 µg/mL) at 24, 48, and 72 h. The PC group, containing CuSO_4_, immensely inhibited the RG at all unveiling times in comparison to the other groups. A low RG profile was observed in the FML-treated groups by increasing the concentration and exposure time compared to the NC. The half-maximal inhibitory concentrations (IC_50_s) of the test groups (FML) were determined for 24, 48, and 72 h, and the values were 0.33 ± 0.12, 0.40 ± 0.04, and 0.34 ± 0.08, respectively. FML at 0.20 µg/mL significantly (*p* < 0.05) inhibited the RG of *A. cepa* at 72 h in comparison to the NC group. However, at 0.40 µg/mL, it was found to exert significant (*p* < 0.05) toxic effects on the roots of *A. cepa* at all of the exposure times.

**Figure 1:**
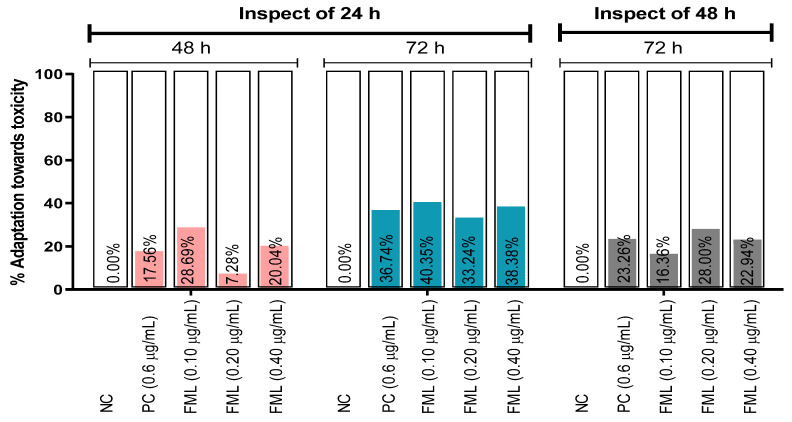
Adaptation towards the toxic effects of the test sample (FML) and controls on *Allium cepa* inspection of 24 and 48 h of exposure time. [Values are a percentage decrease in toxic response in the same group of treatment and an inspection of 24 and 48 h of exposure time. Negative values are omitted in the graph and are represented as 0.00%. NC: distilled water; PC: positive control (CuSO_4_·5H_2_O); FML: formalin].

The percentage of adaptation toward the toxicity of FML has been presented in Figure 1. The result predicts that the highest percentage of adaptation against FML toxicity is 40.35% at 72 h at the lowest concentration of the FML (0.10 µg/mL) inspection of 24 h, but a depletion was observed at 48 h (Figure 1). The result also indicates that the lowest percentage of adaptation toward the toxicity is 7.28% at 0.20 µg/mL at 48 h for 24 h.

Table 2 depicts the average root length of *A. cepa* on an mm scale treated with different concentrations of the artificial sweetener (saccharin) exposed at 24, 48, and 72 h. The highest RG was found at a concentration of 5 µg/mL of saccharin at 24, 48, and 72 h. With the increase in concentration and exposure time of SCN, the inhibition level of RG was elevated, indicating toxicity. The result also demonstrated that there is no percentage inhibition of RG for the concentration of 5 µg/mL for the whole exposed time and for the 155 µg/mL concentration up to 48 h of exposure time. The highest inhibition of RG of SCN was observed at 30.87% for the highest concentration at 72 h of exposure. The half-maximal inhibitory concentrations (IC_50_s) of the test groups (SCN) determined for 24, 48, and 72 h were 33.87 ± 0.16, 34.60 ± 0.15, and 34.32 ± 0.08, respectively. SCN at 15 µg/mL significantly (*p* < 0.05) inhibited the RG of *A. cepa* at 72 h in comparison to the NC group. However, at 25 µg/mL, it was found to exert significant (*p* < 0.05) toxic effects on the roots of *A. cepa* at all of the exposure times.

**Figure 2:**
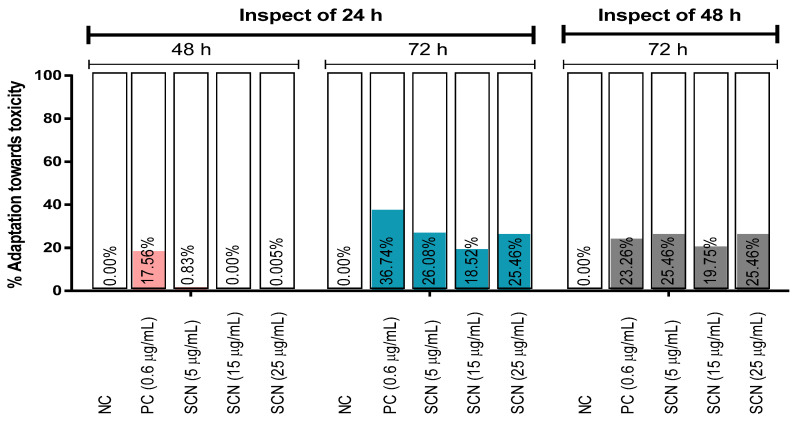
Adaption towards the toxic effects of the test sample (SCN) and controls on *A. cepa* inspection of 24 and 48 h of exposure time. [Values are a percentage decrease in toxic response in the same group of treatment and inspection of 24 and 48 h of exposure time. Negative values are omitted in the graph and are represented as 0.00%. NC: distilled water; PC: positive control (CuSO_4_·5H_2_O)].

The percentage of adaptation towards SCN toxicity has been exhibited in Figure 2. The adaptation was increased against the damage with a time-dependent manner inspection of 24 h. The highest percentage of adaptation (36.74%) was confirmed by the PC at the 72 h inspection of 24 h, whereas no adaptation of SCN at a 15 µg/mL concentration occurred at the 48 h inspection of 24 h, though the percentage of adaptation of the other two groups of SCN was almost negligible at 48 h. On the other hand, 26.08% of adaptation was predicted at 5 µg/mL among all the test concentrations of SCN in the same condition (Figure 2).

In our investigation, *A. cepa* was also treated with different concentrations of urea. In the case of urea, the highest RG of the onions was also seen at the lowest concentration (125 µg/mL) at the maximum exposure time. Table 3 shows the average root length of *A. cepa* in mm after being treated with various concentrations for 24, 48, and 72 h. At 125 µg/mL, no inhibition of RG was observed. The level of inhibition of the sample was increased with the enhancement of concentrations and exposure time. The IC_50_s of the test groups (urea) were also enumerated. The values are 848.10 ± 0.00, 190.10 ± 0.20, and 267.90 ± 0.16 for the exposure times of 24, 48, and 72 h, respectively. Urea at 250 and 500 µg/mL significantly (*p* < 0.05) inhibited the RG of *A. cepa* at 72 h in comparison to the NC group.

**Figure 3:**
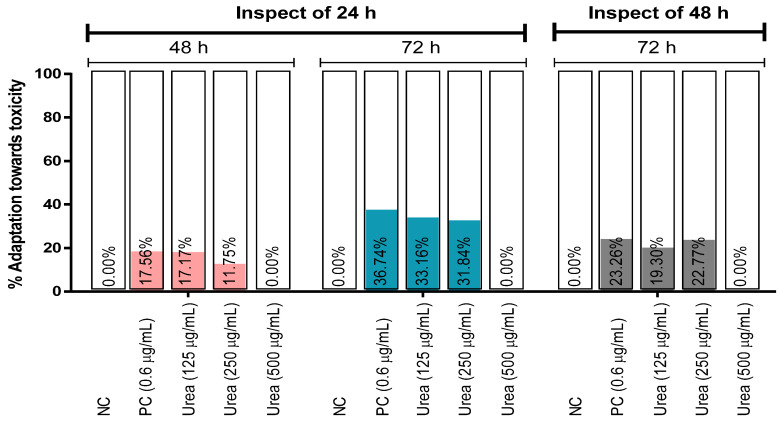
Adaption towards the toxic effects of the test sample (urea) and controls on *A. cepa* inspection of 24 and 48 h of exposure time. [Values are a percentage decrease in toxic response in the same group of treatment and the inspection of 24 and 48 h of exposure time. Negative values are omitted in the graph and are represented as 0.00%. NC: distilled water; PC: positive control (CuSO_4_·5H_2_O)].

Adaptation in percentage against the toxicity of urea on *A. cepa* has been displayed in Figure 3. The results predict that there is no adaptation at the higher concentration of urea, and the highest percentage of adaptation was shown at 72 h (33.16%) at the concentration of 125 µg/mL inspection of 24 h. The results indicate that the percentage of adaptation was gradually enhanced at the 72 h inspection of 24 h. As with the other test samples, the percentage of adaptation was reduced at the inspection of 24 h. The values are displayed in Figure 3.

## 4. Discussion

The assessment of the cytotoxicity and in situ monitoring for genotoxicity of frequently used substances utilizing *A. cepa* is a crucial test system. The assay has been extensively used to investigate the genotoxicity of various chemical compounds, eliciting that these agents can inaugurate chromosomal aberrations (CAs) in the root meristems of *A. cepa* [21]. CAs are specified by an alteration in either the chromosomal structure or in the total number of chromosomes in the root meristems of *A. cepa* due to several factors, such as the inhibition of DNA synthesis, DNA breakdown, and replication of altered DNA from the exposure of different chemical agents (crude extracts) [22,23]. The *A. cepa* test is also capable of the assessment of a number of genetic endpoints, which refer to nuclear abnormalities specified by morphological alterations in the interphasic nuclei [21,24]. The test system is used extensively due to its low cost, easy handling assay, and lack of expensive instruments to evaluate toxicity [5].

The RG profile is a salient feature for realizing the toxic effect of chemical agents in the *A. cepa* assay [25]. A short root length due to the hindrance of RG expresses the toxic effect of the food chemical agents exposed in this assay [26]. Some highly toxic agents, such as the standard (CuSO_4_) utilized in this assay, are manifested to deposit in the roots of *A. cepa* and inhibit RG results from CAs [27]. However, this type of inhibition of RG is typically associated with cell elongation during differentiation [28], apical meristematic activity [29], and the interruption in protein synthesis [30].

In this investigation, CuSO_4_ at 0.6 µg/mL exhibited an increase in the percentage inhibition of root growth (%IRG) for developing an inhibition of RG in a time-dependent manner in comparison to the vehicle. On the other hand, the test chemical agents showed elevated values of the %IRG with an increase in their concentrations. In the case of formalin and saccharin, there was no inhibition at lower concentrations (0.10 and 5 g/mL, respectively), but as the concentration and exposure time increased, the RG was reduced in comparison to the vehicle, with the highest %IRG at 72 h at 0.40 and 25 g/mL, respectively (Table 1 and Table 2). This indicates that the poor root growth of onions at high concentrations due to the action of apical meristematic activity and cell elongation in the process of differentiation [29] expresses genotoxicity. The IC_50_ values of these two chemicals were also gradually increased with the enhancement of exposure time up to 48 h due to the repair and cellular adaption of biological systems with long exposure times [31], but at 72 h, the values were reduced for permanent damage.

In the case of urea, there was no inhibition of root growth at a concentration of 125 µg/mL. On the contrary, the other two concentrations (250 and 500 µg/mL) also exhibited no inhibition up to 48 h; they only showed an inhibition of RG at 72 h in comparison to the negative control (Table 3). The probable reason for no inhibition of RG at lower concentrations and exposure times due to the utilization of urea as a source of nitrogen, which is an essential nutrient, plays an important role in physiological processes [32] because nitrogen is an important element of proteins, enzymes, and vitamins [33]. In some investigations into *A. cepa*, it has been demonstrated that particular chemicals are responsible for CAs in elevated concentrations, but they can exhibit an adaptive response at low concentrations, likely by way of their genomic protective capability [34,35]. For example, in our study, the percentage of adaptation of FML is higher at a low concentration inspection of 24 h of exposure time but at a reduced inspection of 48 h. The results suggest that the cells of the root meristems of *A. cepa* try to recover and adapt against the toxicity of the chemicals as the biological system copes with the new demands entrusted to them by persistently adapting to alterations in the tissue’s circumstance [36]. Not only concentration or dose but also exposure time affect the toxicokinetics and toxicodynamics of toxicants [37]. In this study, we have seen that FML and SCN exerted more toxic effects at their higher test concentrations and at 48 and 72 h of exposure. It has also been confirmed by the highest concentration (500 µg/mL) tested for urea at 72 h, which significantly inhibited the RG profile of *A. cepa*.

Findings from the studies performed using the eukaryotic *A. cepa* test model can be co-related to the laboratory animal and clinical studies. For instance, the toxicogenetic effects of omeprazole at 10, 20, and 40 µg/mL [38] were co-related to the omeprazole-mediated cytogenotoxicological impacts on mouse stomachs, bone marrow, and peripheral blood lymphocytes at 10, 20, and 40 mg/kg [39]. Interestingly, the same test concentrations/doses converted to regular human doses (e.g., 20, 30, or 40 mg) were found to cause genetic instability, cancer [40], and mutagenic effects in humans [41].

## 5. Conclusions

This study showed that the chemical agents (FML, SCN, and urea) used in food processing produced mild concentrations and exposure-time-dependent toxicity in *A. cepa*. With the increase in concentration and exposure time, the %RG was diminished due to chromosomal aberrations in the root meristems of *A. cepa.* The highest %IRG was found in the CuSO_4_ group at 72 h, and the test samples did not exhibit any inhibition of RG at lower concentrations in comparison to the vehicle. The chemical agents exhibited time-dependent protective effects up to the 72 h inspection of 24 h, and a depletion of the %RG was observed at the 72 h inspection of 48 h. Our study suggests that adequate precautions and minimization of doses are needed in the case of industrial and local usage of these chemicals, as the chemicals produced concentration-dependent toxicity in this eukaryotic test system.

## Figures and Tables

**Table 1:** Toxic effects of formalin and the controls on *Allium cepa* root meristems at different exposure times.

Treatments		Root Length (mm)			% Inhibition of RG			IC_50_ (µg/mL) [CI (µg/mL); R^2^]		
		24 h	48 h	72 h	24 h	48 h	72 h	24 h	48 h	72 h
NC		117.80 ± 6.01	185.20 ± 6.19	262.20 ± 3.45	-	-	-	-	-	-
PC		50.00 ± 2.83 *	64.80 ± 4.92 *	70.40 ± 0.97 *	57.55	65.01	73.15	-	-	-
Formalin (µg/mL)	0.10	231.25 ± 4.79	259.25 ± 7.01	307.00 ± 4.45	-	-	-	0.33 ± 0.12 [0.01–10.10; 0.86]	0.40 ± 0.04 [0.12–1.29; 0.98]	0.34 ± 0.08 [0.03–4.11; 0.92]
	0.20	142.00 ± 5.05	207.00 ± 5.14	211.00 ± 3.68 *	-	-	19.527			
	0.40	105.00 ± 4.45 *	132.00 ± 4.92 *	144.00 ± 3.50 *	10.86	28.72	45.08			

Values are mean ± standard error of the mean (SEM) (*n* = 4/5); * *p* < 0.05 when compared to the vehicle (NC) group; one-way ANOVA followed by Tukey post hoc test, considering *p* < 0.05 with a confidence level of 95%; negative values are omitted in the table; NC: negative control; PC: positive control (CuSO_4_·5H_2_O); RG: root growth; IC_50_: half-maximal inhibitory concentration; CI: confidence of interval; R^2^: coefficient of determination at 95% confidence intervals.

**Table 2:** Toxic effects of saccharin and the controls on *Allium cepa* root meristems at different exposure times.

Treatments		Root Length (mm)			% Inhibition of RG			IC_50_ [CI; R^2^]		
		24 h	48 h	72 h	24 h	48 h	72 h	24 h	48 h	72 h
NC		117.80 ± 6.01	185.20 ± 6.19	262.20 ± 3.45	-	-	-	-	-	-
PC		50.00 ± 2.83 *	64.80 ± 4.92 *	70.40 ± 0.97 *	57.55	65.01	73.15	-	-	-
Saccharin µg/mL	05	171.25 ± 5.80	267.00 ± 4.29	281.75 ± 1.36	-	-	-	33.87 ± 0.16 [0.31–3728; 0.89]	34.60 ± 0.15 [0.44–2712; 0.91]	34.32 ± 0.08 [3.10–379.8; 0.97]
	15	120.75 ± 5.19	192.75 ± 4.60	219.00 ± 2.71 *	-	-	16.47			
	25	109.25 ± 3.95 *	171.75 ± 5.40 *	181.25 ± 6.71 *	7.26	7.26	30.87			

Values are mean ± standard error of the mean (SEM) (*n* = 4/5); * *p* < 0.05 when compared to the vehicle (NC) group; one-way ANOVA followed by Tukey post hoc test, considering *p* < 0.05 with a confidence level of 95%; negative values are omitted in the table; NC: negative control; PC: positive control (CuSO_4_·5H_2_O); RG: root growth; IC_50_: half-maximal inhibitory concentration; CI: confidence of interval; R^2^: coefficient of determination at 95% confidence intervals.

**Table 3:** Toxic effects of urea and the controls on *A. cepa* root meristems at different exposure times.

Treatments		Root Length (mm)			% Inhibition of RG			IC_50_ [CI; R^2^]		
		24 h	48 h	72 h	24 h	48 h	72 h	24 h	48 h	72 h
NC		117.80 ± 6.01	185.20 ± 6.19	262.20 ± 3.45	-	-	-	-	-	-
PC		50.00 ± 2.83 *	64.80 ± 4.92 *	70.40 ± 0.97 *	57.55	65.01	73.15	-	-	-
Urea (µg/mL)	125	182.00 ± 7.86	237.00 ± 7.87	270.75 ± 8.18	-	-	-	848.10 ± 0.0 [230.20–3124; 0.99]	190.10 ± 0.20 [0.62–61600; 0.68]	267.90 ± 0.16 [2.61–27556; 0.73]
	250	169.75 ± 7.41	235.50 ± 9.94	257.50 ± 5.59 *	-	-	1.79			
	500	139.50 ± 7.03	198.25 ± 4.43	234.50 ± 8.24 *	-	-	10.56			

Values are mean ± standard error of the mean (SEM) (*n* = 4/5); * *p* < 0.05 when compared to the vehicle (NC) group; one-way ANOVA followed by Tukey post hoc test, considering *p* < 0.05 with a confidence level of 95%; negative values are omitted in the table; NC: negative control; PC: positive control (CuSO_4_·5H_2_O); RG: root growth; IC_50_: half-maximal inhibitory concentration; CI: confidence of interval; R^2^: coefficient of determination at 95% confidence intervals.

## Data Availability

The processed data are available from the corresponding author upon request.
